# Automation Technology and Sense of Control: A Window on Human Agency

**DOI:** 10.1371/journal.pone.0034075

**Published:** 2012-03-30

**Authors:** Bruno Berberian, Jean-Christophe Sarrazin, Patrick Le Blaye, Patrick Haggard

**Affiliations:** 1 Systems Control and Flight Dynamics Department, ONERA, Toulouse, France; 2 Institute of Cognitive Neuroscience, University College London, United Kingdom; Royal Holloway, University of London, United Kingdom

## Abstract

Previous studies have shown that the perceived times of voluntary actions and their effects are perceived as shifted towards each other, so that the interval between action and outcome seems shortened. This has been referred to as ‘intentional binding’ (IB). However, the generality of this effect remains unclear. Here we demonstrate that Intentional Binding also occurs in complex control situations. Using an aircraft supervision task with different autopilot settings, our results first indicated a strong relation between measures of IB and different levels of system automation. Second, measures of IB were related to explicit agency judgement in this applied setting. We discuss the implications for the underlying mechanisms, and for sense of agency in automated environments.

## Introduction

### Automation and feeling of control

We live in an increasingly technological world. Automated systems certainly can make life easier, but can also create complexity and uncertainty. For example, the important role of automation aids in aviation can lead pilots to the following important question: “who is in control now?” [1]. More particularly, the interposition of more and more automation between pilot and aircraft tends to distance pilots from many details of the flying, decreasing their feeling of control [2]. Measuring the feeling of control may be important in evaluating different automated devices, and may also be relevant to evaluating pilot performance. It follows that better measurement and psychological understanding of the human sense of control could contribute to better automatic system design. On the other hand, studying complex control situations, like human interactions with automated devices, may improve our understanding of cognitive mechanisms that underlie the feeling of control.

### Quantifying human agency

When we act, we usually have a clear feeling that we control our own action and can thus produce effects in the external environment. This feeling has been described as “the sense of agency” [3], and is recognised as an important part of normal and human consciousness. However, the sense of agency has proved difficult to quantify, and its basis and limits remain unclear. Central to the sense of agency is temporal contiguity between one's action and the resultant effects [4]–[6]. Interestingly, the reverse seems also true: human intentional actions produce systematic changes in time perception. In particular, the interval between a voluntary action and an outcome is perceived as shorter than the interval between a physically similar involuntary movement and the same outcome event. This phenomenon, called intentional binding [7], may provide an implicit window into human agency. Intentional binding has been widely reported [8]–[14].

Several questions remain about the factors that produce a sense of agency. For example, agency comes by degrees: one can feel more or less in control. This variation is particularly clear when using machines. The feeling of control varies quite subtly as the relation between operator inputs and machine response [2], [15], [16]. However, previous tasks relied either on explicit binary judgements of agency vs. non-agency in self-other discrimination paradigms [17], or on contrasting binding between entirely voluntary and entirely involuntary situations [7]. Finally, interactions with complex machinery are clearly one area where sense of agency is important, but may be difficult to achieve: surprisingly no study, as far as we know, has investigated sense of agency in such applied settings.

We therefore explored sense of agency in a complex setting involving flying an aircraft with various degrees of autopilot assistance. Using both implicit (intentional binding) and explicit (verbal reports) measures of agency, we demonstrate that degree of automation influences both measures. Our results validate intentional binding as a measure of sense of agency, identify the conditions for experiencing agency in automation settings, and suggest new measures for quantifying human experience of control over critical machinery.

## Methods

### Ethics Statement

All participants signed a written declaration of informed consent. The procedure was conducted in accordance with the Declaration of Helsinki, but no specific ethical approval was obtained considering that ONERA has no Institutional Review Board.

### Participants

Thirteen participants (4 females; mean age of 32 years) from the french aerospace lab took part. Participants were naive to the purposes of the manipulation, had normal or corrected- to-normal vision and no particular expertise in controlling an aircraft.

### Materials and apparatus

**Figure 1:**
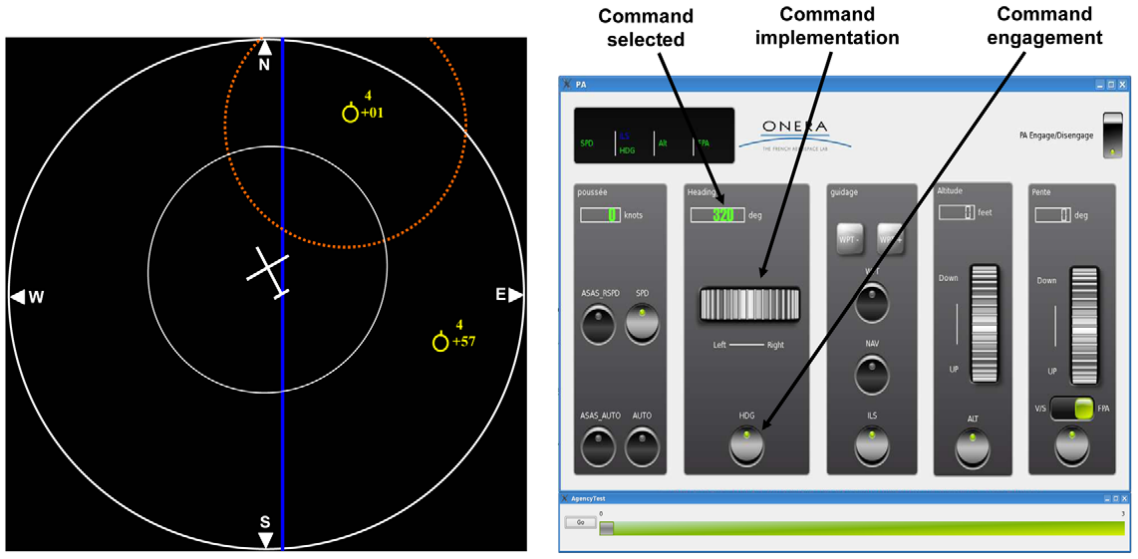
Experimental set up with the navigation display on the left, and the autopilot interface on the right.

The simulator (see Fig. 1) included a navigation display (22-in screen) including the navigation of the aircraft in the horizontal plane and the surrounding traffic. An adjacent monitor (17-in touch screen) showed an autopilot interface, and allowed the participant to change the aircraft's horizontal trajectory in case of conflict, such as being too close to another aircraft. Moreover, a scale below the autopilot interface enabled recording of participants' responses to agency questions (see later). Thus, the autopilot interface allowed the participant to choose and then execute actions, in a manner analogous to the keypresses in a standard agency experiment. The navigation display showed the effect of the executed action as a visual representation of aircraft heading.

### Design and procedure

The participants' task was to track the progress of their aircraft on a predefined flight path and intervene as the situation required. In particular, when another aircraft appeared on the flight path, the participant had to perform an appropriate command (see later) to change their aircraft's heading direction using the autopilot interface.

**Figure 2:**
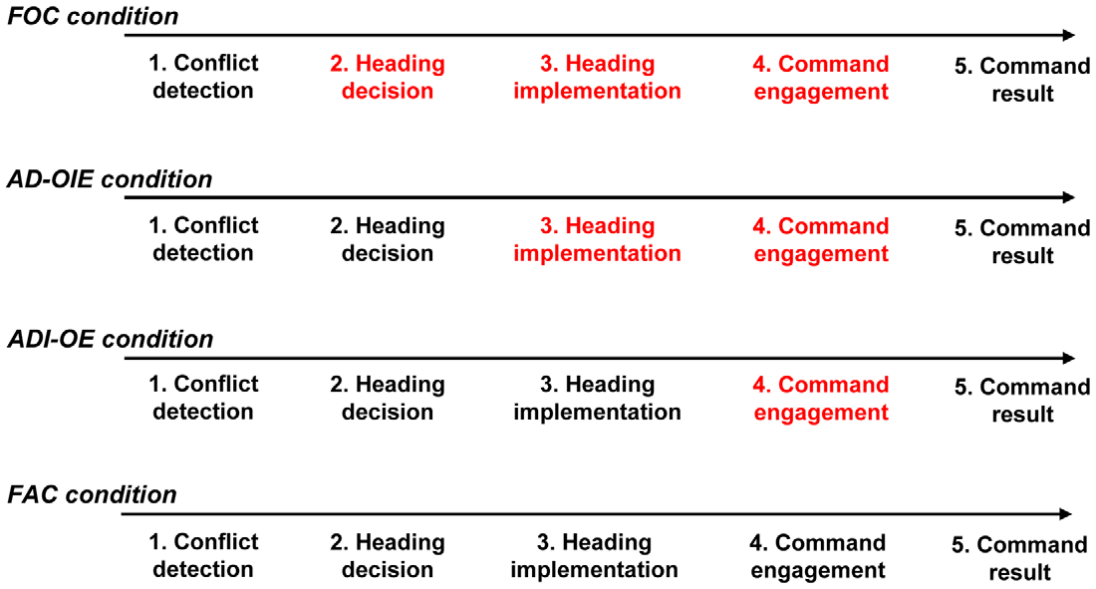
Typical sequence of events for one trial.

The sequence of events on each trial was as follows (see Fig. 2). At the beginning of each trial, participants supervised the navigation of the aircraft on the flight plan. (1) After a randomized short interval, a conflict appeared due to presence of an obstacle (another aircraft) on their path. The participant detected the conflict by a red circle around the obstacle. (2) The participant decided an appropriate heading command, (3) implemented it using a scroll wheel on the interface, and (4) finally executed it by pressing an engagement button. Importantly, the action was effective only after the participant's engagement of the command. This engagement was marked by the appearance of a green light on the interface coupled to a short sound. (5) After a controlled temporal delay, feedback concerning the success of the action engaged was sent to the participants: a green message “resolved” plus a sound if successful action; a red message “not resolved” with a different sound if not. (6) After each trial, and whatever the success of the command engaged, participants had to estimate on a scale from 0 s to 3 s the temporal delay perceived between the keypress to engage their command and the appearance of the visual feedback (“conflict resolved” or “conflict not resolved”). In the Full Automated Control condition (see below), where participants did not make a keypress, they judged the time between the appearance of the green ‘engagement’ light plus accompanying sound and the conflict resolution feedback. They were told that the possible range of delays was between 1 ms and 2999 ms. In fact, only three Action/Effect delays (750 ms, 1500 ms, and 2250 ms) were presented, in a random order.

**Figure 3:**
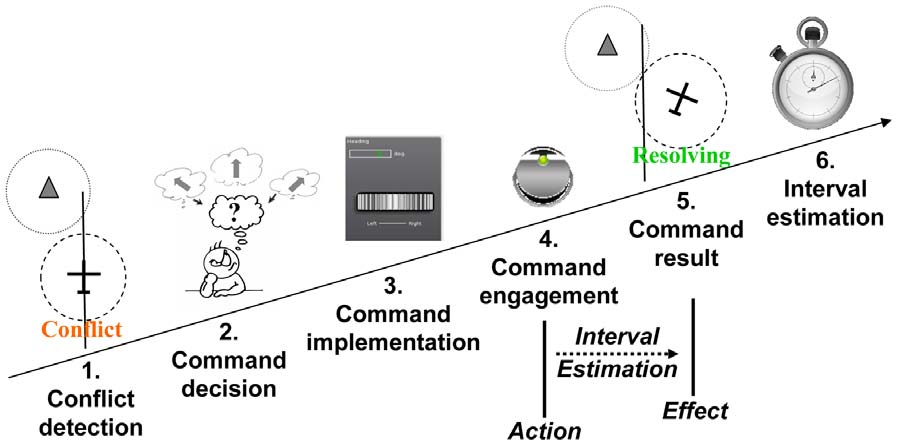
Automation level and cognitive processes in our aircraft navigation task. The red text indicates functions performed by the human operator and black text indicates function performed automatically by the system.

In order to meet the task requirement, participants had automation tools to predict conflicts with the surrounding traffic, alert humans of this conflict, and provide commands or guidance to resolve the conflict. In order to study how level of automation affect the sense of agency, automation level was varied between block of trials. In accordance with an established classification [18], four different levels of automation were tested (see Fig. 3) with, from the least automated condition to the more automated condition:

The *Full Operator Control (FOC)* condition: conflict detection was automatic; Heading decision, implementation of the decision, and engagement of the adjusted heading command were performed by the participant;
The *condition*: conflict detection and heading decision were automatic (navigation display indicated a new heading direction which would avoid the conflict); Implementation of the indicated heading using the scroll wheel, and engagement of this decision using the keypress were performed by the participant;
The *Automatic decision and implementation - Operator engagement (ADI-OE) condition*: conflict detection, heading decision and implementation of this decision were automatic; Engagement of the command was performed by the participant with a keypress;
the *Full Automatic control (FAC)* condition: Automation tools predicted the conflict, selected, implemented and engaged the adapted command (indicated by a sound coupled to a green light). The operator merely observed.

Each participant performed four blocks of trials, each block of trials corresponding to one level of automation. The blocks were tested in random order. Within each block of trials, participants experienced 9 trials (three for each Action/Effect delay tested, again in random order). Finally, at the end of each block of 9 trials, participants made an explicit judgement of agency, by verbally reporting how strongly they felt that they caused the manoeuvre to avoid conflict, using a scale from 0 (no causal involvement) to 3 (strong causal involvement).

## Results

In this study, our primary concern is the relationship between level of automation and our two measures of sense of agency: the perceived duration of intervals between actions and effects and the explicit judgement of causal control.

### Temporal judgement

Each participant made 3 temporal judgments for each combination of *Automation Level* and *Action/Effect delay*. Participants' mean temporal judgments served as the primary units of analysis. We performed a 4* 3 ANOVA with *Action/Effect delay* (750, 1500, 2250 ms) and *Automation level* (*FOC, AD-OIE, ADI-OE* and *FAC*) as within subject factors.

**Figure 4:**
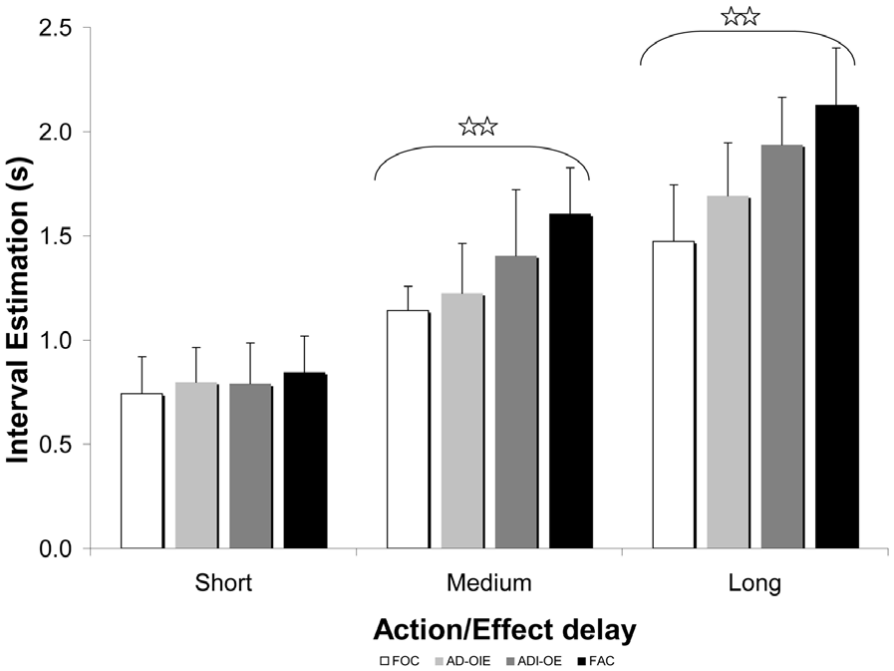
Modulation of interval estimates by actual interval between action and effect for each automation level. Stars represent significant effects (p<.01).

There was a significant main effect of *Action/Effect delay*, F(2,24) = 209.68; p<.01., η_p_^2^ = .95 (see Fig. 4). Post-hoc analysis revealed that the interval estimates increased monotonically with the actual interval: the longer the actual action delay was, the longer the action-effect interval was perceived (all *p*s<.01). These results indicate that participants were able to track the physical variation of the interval.

**Figure 5:**
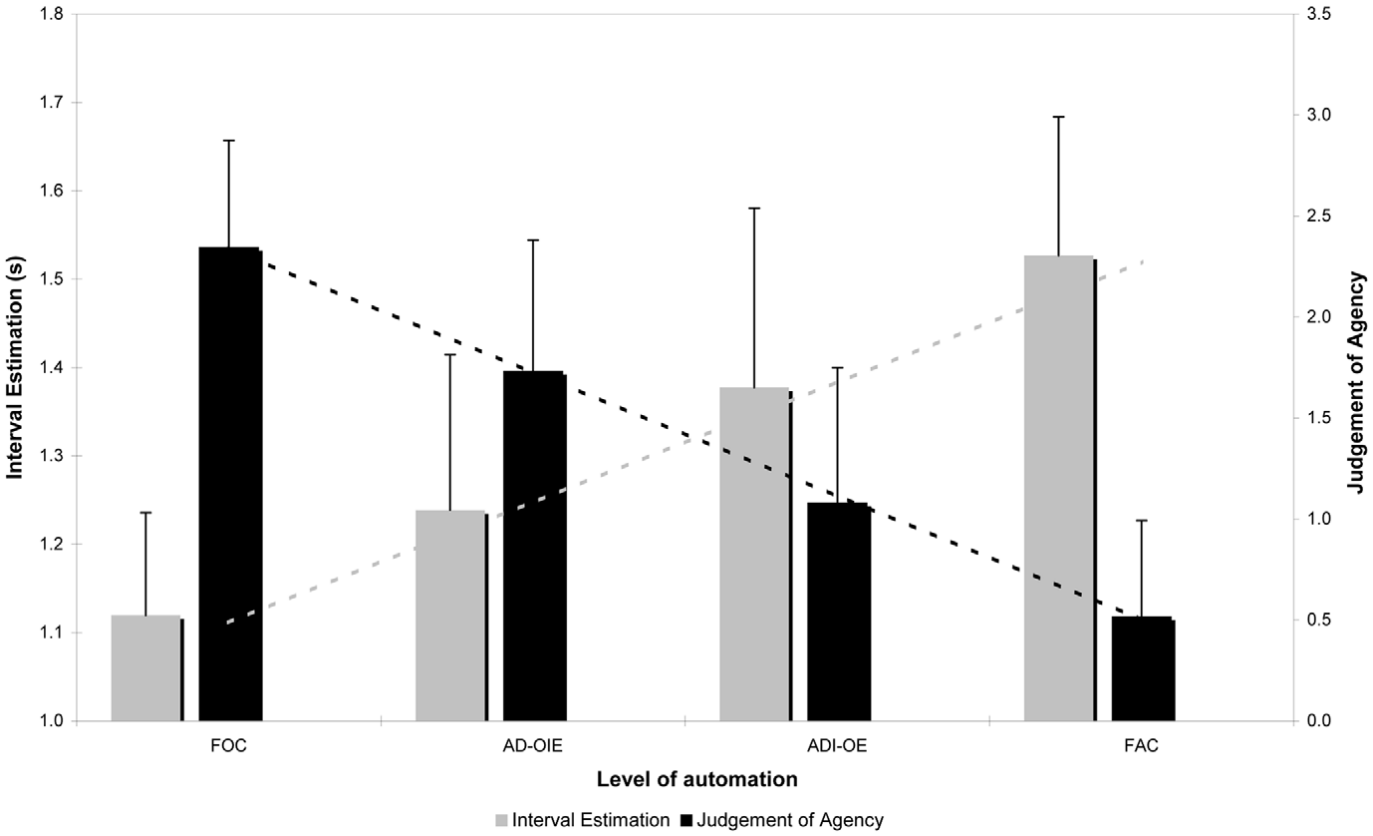
Modulation of interval estimates and explicit judgement of agency by automation level.

More interestingly, there was also a significant main effect of *Automation level*, *F*(3,36) = 26.154; *p*<.01, η_p_^2^ = .69 (see Fig. 5). Post-hoc analysis revealed that interval estimates increased monotonically with the level of automation: the more the system was automated, or the less it relied on participant's actual control, the longer the action-effect interval was perceived (all *p*s<.01). These results indicate that IB is sensitive to levels of automation, with increasing automation leading to a higher interval estimates, which we interpret as a gradual decrease in sense of agency.

Finally, the effect of Automation Level was modulated by the Action/Effect delay, as demonstrated by the significant two-way interaction between these factors: *F*(6,72) = 8.457; *p*<.01, η_p_^2^ = .42. Post-hoc analysis reveals that interval estimates from the medium and large Action/Effect delays were strongly modulated by the automation level (*p*<.01), whilst estimates from the small Action/effect delays were not significantly modulated (*p*>.01) (see Fig. 4).

### Explicit Judgement of Agency

Each participant returned one judgment of agency per Automation Level (see Fig. 5). A repeated measures ANOVA showed a significant influence of Automation Level, *F*(3,36) = 46,204; *p*<.01, η_p_^2^ = .79. Post hoc analysis shows that judgement of causality decreased monotonically with the level of automation (all *p*s<.01). As expected, explicit judgements of agency follow the facts of agency. But this is not completely trivial, as judgements of agency frequently do not follow actual control in situations where agency is ambiguous [19], [20].

Finally, we correlated the mean binding effect and the explicit judgement of agency across the four levels of automation for each individual subject. We then tested the resulting *r*(3) correlation coefficients for the group of all 13 subjects against 0. Correlation between authorship and binding effects was negative and significant (Mean r = −0.84, SD = 0.105, *t*(12) = −28.821, *p*<.001), indicating that as actual levels of control were varied, changes in intentional binding closely tracked explicit judgements of agency. This finding boosts the use of intentional binding as an implicit window into human agency.

## Discussion

In this experiment, we explored intentional binding in a rich and complex situation involving flying an aircraft with various degrees of autopilot assistance. Our study yielded three important results. First, we observed a strong relation between measures of IB and different levels of system automation. Second, our data revealed a gradual increase in temporal estimation with the increasing level of automation. The more the system was automated, the longer the action-effect interval was perceived. Third, the effect of automation level on intentional binding was dependent on the actual action-effect delays. There was a strong effect of automation level on binding for medium and large action-effect intervals and no effect for small action-effect intervals. Such findings have important implications concerning the precise conditions under which the intentional binding effect occurs: intentional binding is an empirically robust phenomenon that occurs in complex control situations, is sensitive to graded variations in actual level of control, and is task dependent. We now discuss these in turn.

### Robustness of the binding effect

Our findings confirm the interest of the intentional binding as implicit measure of agency. First, we provide evidence that quantitative changes in binding are strongly associated with progressive changes in actual level of control, and also with quantitative changes in explicit ratings of agency (but see [9]). Second, we replicate the basic binding effect in a situation with high face-validity, in which action-event sequences paralleled those that participants might meet in their everyday lives. Interactions with machines regularly involve sending a command to a system, and monitoring the system response, and we regularly feel a sense of controlling how the machine behaves in such situations. Our data thus lend external validity to intentional binding. They confirm that temporal distortions associated with agency occur in everyday life. Intentional binding may be a useful measure in future cognitive engineering studies.

### Binding by degrees

Our results reveal a gradual increase of interval estimates with the increasing level of automation. In contrast, many previous studies of agency have relied on binary agency-attribution judgements [21]. In binary agency-attribution, judgements of agency may depend on a match between predicted and actual effects, leading to a simple ‘me’ vs. ‘not me’ decision [22]. Our finding of binding by degrees goes beyond a simple compare and decide model. Instead, our results suggest that several features of the way in which intentions guide and sustain action contribute to the experience of acting, and the sense of control. In particular, in our task participants always judged the interval between command engagement and conflict resolution. However, we found that the amount of operator involvement in processing stages *preceding* the command engagement, such as decision and implementation of the heading adjustment, strongly influenced interval estimates. Thus, specific subprocesses of intentional control may be relevant for sense of agency. For example, processes that generate and select between action alternatives may also contribute to the sense of control over action outcomes (see also [23]). In contrast, previous work emphasised the role of retrospective *comparison* of intended and actual outcomes in agency judgements [24], [25]. More generally, our result suggests action selection, planning and intention realisation may all contribute to sense of agency. In this way, our results are consistent with recent arguments that sense of agency is based on cue integration [26].

### Binding effect and time constraints

Our results suggest that temporal binding is not present for the shortest interval tested here (750 ms). Other studies have suggested that longer action–outcome intervals were associated with reduced binding [7], [9], [27]. Here we show that very short intervals could also decrease the intentional binding effect. Interestingly, the short interval tested here corresponds to a large interval in previous studies [9], suggesting this is unlikely to be simply a floor effect. The complex nature of the actions and their effects in our device may explain the preferential binding over longer intervals. We propose that temporal contiguity is task dependent and that intentional binding occurs in a specific “window of opportunity” which may vary across tasks, and may also depend on the range of action-effect delays experienced in a given setting. Operant learning is similarly sensitive to the natural time delays of the system linking actions to effects, even for systems as familiar as one's own body. For example, when rats learn to avoid eating food associated with illness, the optimal delay between eating and illness is not the shortest possible delay, but a delay consistent with their normal digestive operation [28].

### Conclusion

We found a distortion of time perception in the control of complex machinery, which adds to a growing literature on sense of agency. Our findings are significant in four ways: First, demonstration of binding effects in a richer and more complex paradigm provides external validity for intentional binding. Second, we show agreement between implicit binding measures of sense of agency, and explicit agency judgement, in such a complex setting. Third, we demonstrate that intentional binding occurs over temporal windows between action and effect that are longer than those studied previously (see also [11], [29]), according to the significant features of the given task. Fourth, we show that intentional binding measures are sensitive to graded variations in actual and subjective control associated with automation.

The link between automation and sense of agency holds promise for future applications. From a cognitive engineering viewpoint, the ability to measure sense of agency quantitatively is important, since it allows sense of agency to be used as a measure in evaluating operator experience. In future research, we will test whether systems that produce stronger subjective sense of agency also produce better performance. When we get on an airplane, we believe (and hope!) that the pilot feels in personal control of the aircraft. Our results offer the interesting possibility of testing whether this is actually true. In a next step, our work could provide guidelines regarding how to boost this feeling of control, and could assess whether feeling of control are related to actual levels of performance in controlling the aircraft.
